# The Diagnostic Value of CT Scan in Identifying Adenoid Hypertrophy in Adults: A Case Report

**DOI:** 10.7759/cureus.62793

**Published:** 2024-06-20

**Authors:** Sudhanshu Tonpe, Himandri Warbhe, Pankaj Banode, Sohini Gandham, Vadlamudi Nagendra

**Affiliations:** 1 Department of Interventional Radiology, Jawaharlal Nehru Medical College, Datta Meghe Institute of Higher Education and Research, Wardha, IND; 2 Department of Respiratory Medicine, Jawaharlal Nehru Medical College, Datta Meghe Institute of Higher Education and Research, Wardha, IND; 3 Department of Radiology, Apollo Institute of Medical Sciences and Research, Hyderabad, IND; 4 Department of Radiology, Jawaharlal Nehru Medical College, Datta Meghe Institute of Higher Education and Research, Wardha, IND

**Keywords:** ct scan, snoring, mouth breathing, nasal obstruction, adult adenoid hypertrophy

## Abstract

The utility of computed tomography (CT) scan in diagnosing adenoid hyperplasia in adults. A 22-year-old woman presented with persistent bilateral nasal obstruction, anterior nasal discharge, mouth breathing, and snoring over the past three months. Despite attempts with both local and systemic decongestants, there was no improvement, and flexible nasopharyngoscopy could not be conducted. CT scans revealed a heterogeneously enhancing space-occupying mass in the nasopharynx, and a rare diagnosis of adult adenoid hypertrophy was reported. The patient responded to a combination of painkillers, antibiotics, and nasal decongestants.

Adenoid hyperplasia in adults is quite rare and inadequate examination by indirect posterior rhinoscopy may lead to misdiagnosis and mismanagement. A CT scan not only provides a clearer view of the nasopharyngeal space and adenoids but also reveals details about the nature of lesions, including their extension and potential bone destruction, suggesting the presence of a malignant tumor. Additionally, a CT scan proves valuable in diagnosing chronic sinusitis.

## Introduction

Adults rarely experience adenoid hyperplasia. It is usually inadequately examined by indirect posterior rhinoscopy, leading to misdiagnosis and maltreatment [1]. In adults, adenoid hypertrophy may result from persistent childhood adenoids linked to chronic inflammation, renewed growth in response to infections and irritants, and compromised immunity due to underlying factors, such as organ transplant recipients or individuals with human immunodeficiency virus (HIV) infection [1].

It is possible for enlarged adenoids to totally obstruct nasal passageway ventilation. If they are not significantly enlarged, they can impede airflow to a degree that nasal breathing becomes laborious, leading to discomfort, mouth breathing, alterations in voice, sleep apnea, and snoring.

## Case presentation

A 22-year-old female patient is described in our case study. She had been experiencing significant bilateral nasal obstruction, mouth breathing, anterior nasal discharge, and snoring for the previous three months, and neither local nor systemic decongestants helped.

**Figure 1:**
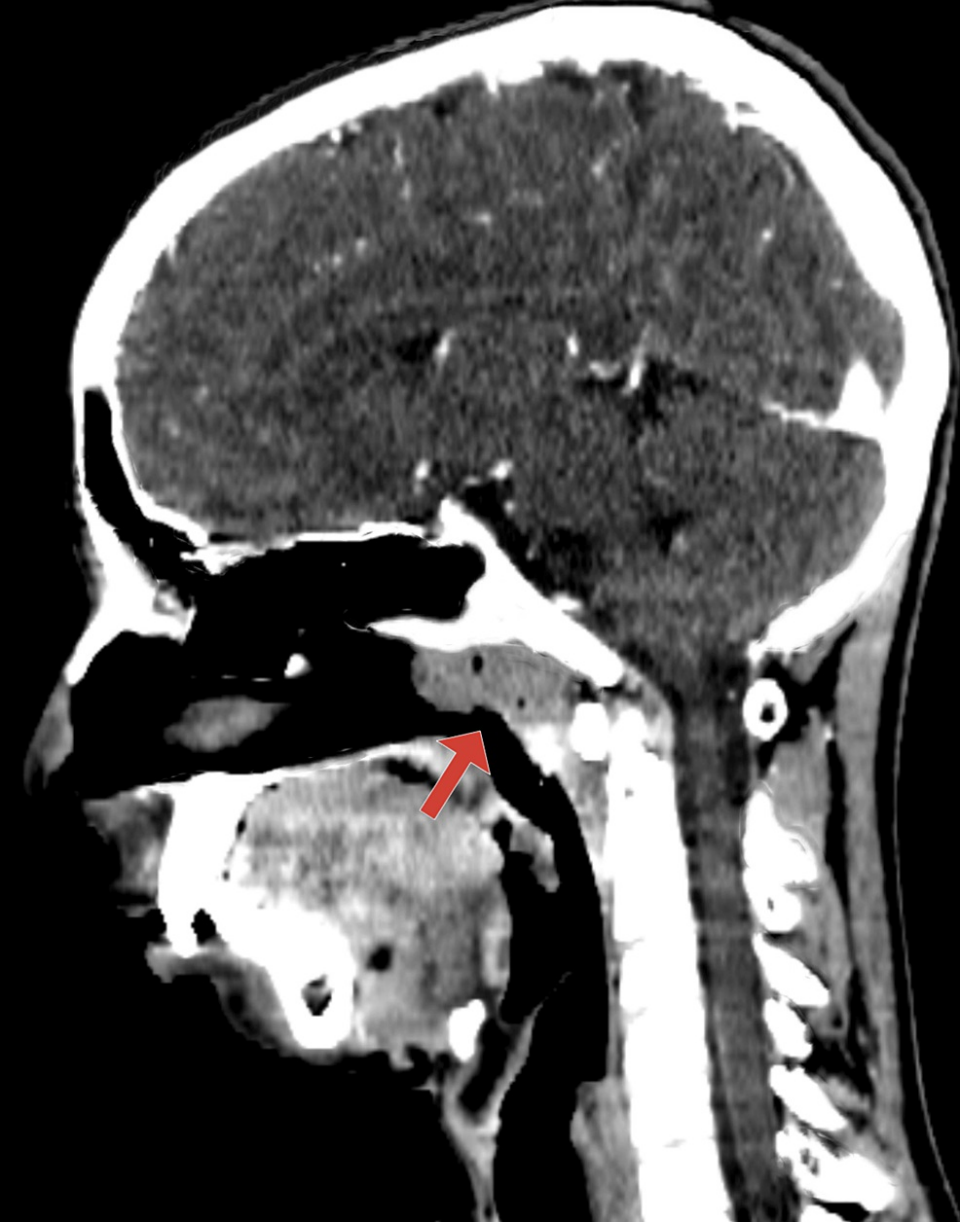
CT demonstrates a heterogeneously enhancing mass (red arrow) occupying the nasopharynx

Upon examination, a purulent discharge filled the nostril. Flexible nasopharyngoscopy could not be carried out. Scans using CT showed that the nasopharynx was occupied by a heterogeneously enhancing mass (Figure 1).

The CT appearance of the lesion was diagnostic of hypertrophied adenoid; hence no further imaging investigation was performed.

The patient was treated conservatively with antibiotics that included Amoxicillin beta-lactamase inhibitor clavulanic acid, nasal decongestants, and painkillers for symptomatic and curative relief. No interventional was indicated.

## Discussion

Adenoid is the condensation of lymphoid tissue at the postrosuperior wall of the nasopharynx. It constitutes a component of Waldeyer's Ring, playing a role in safeguarding the body against the infiltration of bacteria, viruses, and toxins. It is important in developing an "immunological memory" in younger children [2]. The adenoid and tonsillar tissues predominantly consist of a cluster of blood cells known as B lymphocytes, responsible for antibody production. These antibodies attach to viruses, bacteria, and toxins, rendering them inactive and preventing their entry into the body, thereby averting the onset of disease.

Physiologically, adenoid hypertrophy naturally occurs in children aged six to 10 and subsequently undergoes atrophy by the age of 16 years [3]. Unlike the tonsils, which protrude directly through the mouth, the adenoid extends upward behind the soft palate and is located at the farthest posterior end of the nasal cavity. Similar to tonsillar tissue, infections, both acute and chronic, can cause the adenoid to gradually enlarge.

Age-related changes as evaluated through CT, MRI, and PET reveal a notable reduction in adenoid size as an individual age. Despite adenoid tissue regressing during adolescence, adenoid hypertrophy is observed in the normal adult population as well [4,5]. Adenoid hyperplasia in adults is quite rare and inadequately examined by indirect posterior rhinoscopy, leading to misdiagnosis and maltreatment [1]. While the exact cause of adenoid hypertrophy is uncertain, some theories have been put up.

Enlarged adenoids in adults can stem from the continued presence of childhood adenoids linked to chronic inflammation, renewed growth triggered by infections and irritants, and compromised immunity, particularly in individuals undergoing organ transplants or those with human immunodeficiency virus (HIV) infection.

Adenoid hypertrophy presents with diverse clinical features. Nasal obstruction, experienced by all patients, may lead to mouth breathing, recurrent nasal infections, and hyponasal speech [1,6]. Children with adenoid hypertrophy are reported to have a higher incidence of snoring compared to adults [3].

Studies have found persistent infection with *Hemophilus influenza*, a normal upper respiratory tract bacterium, in comparison with adenoids of a more typical size. Adenoid hypertrophy can also be brought on by long-term allergies and discomfort from infected or inflammatory nasal secretions that are brushed back over the area.

Adenoidal hyperplasia is a rare occurrence in adults, and its identification raises concerns about potential malignancies, such as type B white blood cell (lymphoma plasmacytoma) or HIV complications, common in the head and neck regions [7]. Many lesions within the oral cavity related to HIV infection cause intense pain [8-10].

Persistent adenoidal enlargement can result in chronic mouth breathing and ear diseases. There is apprehension that prolonged mouth breathing in children may contribute to facial abnormalities, including mid-face elongation and a narrow, high-arched palate, leading to orthodontic issues. Undiagnosed obstructive sleep apnea linked to enlarged adenoids can result in severe outcomes, such as pulmonary hypertension, reduced mental alertness, and enlargement of the right side of the heart [8-10].

Antibiotics and oral steroids are often effective in managing acute conditions of enlarged adenoids. For some individuals, long-term nasal steroid sprays may help reduce adenoid size. Surgical intervention is commonly recommended for cases that do not respond to these medical approaches [10].

## Conclusions

Considering the rarity and potential dangers associated with adenoid hyperplasia in adults, including its links to lymphoma, malignancies, and occasionally HIV infection, early diagnosis is crucial. With prompt treatment, the prognosis can be favorable. Hence, it is imperative not to disregard any suspected case of adult adenoid hypertrophy, and a CT scan is recommended for a thorough evaluation and timely intervention. 
